# The highest Duffy (*FYX*) allele frequency ever reported for Scottish population: A cross‐sectional study

**DOI:** 10.1002/hsr2.1314

**Published:** 2023-06-02

**Authors:** Sylvia Armstrong‐Fisher, Stan Urbaniak, Arian Karimi Rouzbahani, Mona Hemmati Chegeni, Golnaz Mahmoudvand, Ali Mohammad Varzi

**Affiliations:** ^1^ Division of Applied Medicine, Institute of Medical Sciences University of Aberdeen Aberdeen UK; ^2^ Student Research Committee Lorestan University of Medical Sciences Khorramabad Iran; ^3^ USERN Office Lorestan University of Medical Sciences Khorramabad Iran; ^4^ Department of Immunology Lorestan University of Medical Sciences Khorramabad Iran

**Keywords:** BeadChip microarray, Duffy blood group, FYX allele, Scottish population

## Abstract

**Background and Aim:**

The Duffy (FY) blood group system has six known antigens among which the Fy^a^ and Fy^b^ are known as major antigens. Fy^x^ phenotype forms as a result of two point mutations in the *FYB* allele leading to instability of Duffy protein and so reduction of Duffy antigen expression in the cells. This study aimed to investigate the *FYX* allele frequency in the Scottish population.

**Methods:**

The Duffy blood group system was serologically and molecularly investigated in 222 samples collected from donors of Aberdeen Regional Blood Transfusion Center (BTC). The haemagglutination and BeadChip microarray chemistry methods were used for phenotyping and genotyping. Confirmatory tests were also used to check the discrepant results.

**Results:**

In this study, the frequency of Duffy blood group phenotypes including Fy^a+^, Fy^a+b+^, and Fy^b+^ were 17.57%, 42.79%, and 39.64%, respectively. Furthermore, the frequency of *FYA/FYA*, *FYA/FYB*, and *FYB/FYB* genotypes was estimated to be 14.41%, 45.95%, and 39.64%, respectively, using the Bioarray method. In the present study, based on Duffy DNA sequencing results, 12 samples (5.41%) had just one *FYX* allele.

**Conclusion:**

The frequency of the *FYX* allele in this study was estimated to be 0.0270% which is more than the results reported so far.

## 1 INTRODUCTION

The Duffy blood group system is a clinically remarkable blood group system.^1^ It comprises six antigens (Fy^a^, Fy^b^, Fy^3^, Fy^4^, Fy^5^, Fy^6^), and four main phenotypes; Fy(a+b+), Fy(a−b+), Fy(a+b−), and Fy(a−b−).^2^, ^3^ The antigens are expressed on a glycosylated erythrocyte membrane protein known as the Duffy antigen receptor for chemokines (DARC). The absence of DARC on the red blood cell surface is correlated with various conditions such as inflammation, human immunodeficiency virus infection, and malignancies. Duffy blood group negativity is prevalent among Africans causing erythrocytes resistance to invasion by *Plasmodium vivax* and *Plasmodium knowlesi*.^4^ In 1950, Fy^a^ was discovered after finding anti‐Fy^a^ in the serum of a hemophilic person who had received several transfusions.^5^ One year later, after finding anti‐Fy^b^ in the serum of a woman who had been pregnant three times, Fy^b^ was discovered.^6^ The Fy^x^ phenotype is a weak form of Fy^b^ that was reported later by two other groups.^7^, ^8^ The *FYX* allele has a frequency of 2%–3.5% in Caucasian people while it is not seen in Blacks.^9^ *FYA and FYB* alleles differ by a single G to A nucleotide substitution at position 125 in exon 2.^10^ The molecular basis of the Fy (a‐ b^−^) phenotype is related to the mutation in the GATA promoter region of the *FYB* (−67, T to C). Disturbance at the binding site for the GATA‐1 erythroid transcription factor can be considered a result of this nonsense mutation.^9^ Moreover, a similar mutation is observed in the GATA‐1 promoter region of the *FYA*.^11^ The highest frequency for Fy^b^ was observed when a group studied the frequency of Fy^a^ and Fy^b^ in Blacks using the polymerase chain reaction with restriction fragment length polymorphism (PCR‐RFLP) method.^12^ Owing to the high incidence of discrepancies between Fy^b^ phenotyping results versus genotyping outcomes on the collected samples from Scottish blood donors through the PCR‐RFLP method, Murphy suggested that the frequency of Fy^x^ in Caucasians was more than the previously reported 0.015%.^13^, ^14^ To determine the pregnancies at risk for HDFN, Hessner et al. used a validated PCR‐ASP assay for prenatal genotyping in 1999. The method utilized was able to determine the *FYA, FYB*, null *FYA*, and null *FYB* alleles.^15^ One year later, the two missense mutations related to *FYX* were defined using the PCR with sequence‐specific primers (PCR‐SSP) method in 300 samples.^16^ Schmid and co‐workers reported a frequency of 1% for Fy^x^ haplotypes in 54 samples of African American blood donors in 2011.^17^ In 2015, Silvia Manfroi and co‐workers used the BLOODchip® *IDcor* ^*+*^ technique to determine the *FYX/FY* null and *FYA/FY* null genotypes in a Caucasian thalassemia family from Sardinia and investigated the *FYX* and *FY* null alleles frequency in Caucasian and black people.^9^ In the present study, the prevalence of the *FYX* allele was measured in a Scottish population using high‐sensitivity molecular assays.

## 2 MATERIALS AND METHODS

To study the Duffy blood group serologically and molecularly, blood samples were taken from 222 blood donors. Forty‐seven of the donors (male = 19, female = 28) were healthy and partially known reference panel donors of Aberdeen Regional Blood Transfusion Center (BTC), and the other blood samples were taken from active, healthy platelet donors (*n* = 175, male = 126, female = 49) who consented to participate in the study. The collection of blood samples began after the project was approved by the Grampian Local and Regional Ethics Committee (LREC) and the Scottish National Blood Transfusion Services (SNBTS) Clinical Governance committee. The purified genomic DNA (gDNA) of buffy coat from whole blood EDTA samples was genotyped by BeadChip microarray chemistry, that is, BioArray HEA *BeadChip™* and their red blood cells were used for phenotyping. The commercial kits, READY GENE KKD PCR‐SSP, in‐house DNA‐PCR SSP assay, and serological methods were used for re‐genotyping and re‐phenotyping due to the existence of discrepant results. The mentioned methods are overviewed in the section below.

### 2.1 BeadChip microarray

Microarray‐based technologies are known as powerful techniques to study gene expression patterns on a genome‐wide scale.^18^, ^19^, ^20^ Recently, the human erythrocyte antigen (HEA) BeadChip array of bioarray solutions, which has been used in the present study to investigate the samples, has become more popular as microarray chemistry. In our study, the HEA24 and HEA28 BeadChip kits were used for genotyping of donor samples. The following five steps are necessary to use these kits; multiplex PCR amplification of target markers on the gDNA, cleaning up of the PCR products using ExoSAP‐IT, generating single‐strand DNA (ssDNA) products using Lambda Exonuclease, hybridization/and elongation of the ssDNA to the specific probes on the BeadChips and finally reading the images using a Microarray AIS400 system (Bioarray Solutions) and analyzing the data online.^21^

The results of allele frequency, allele detection rates (ADR), corrected allele detection rate (CADR), and concordance rates (CoR) were calculated based on the following formula, respectively:

(1) $$F\left(\%\right)=\frac{n\times AA+\left[\left(n\prime ÷2\right)\times AB\right]}{N\times 2}\times 100,$$

where *n*, *AA*, *n*ʹ, *AB*, and *N* denote the number of detected homozygous, homozygous, the number of detected heterozygous, heterozygous, and total sample number, respectively.

(2) $$ADR\left(\%\right)=\frac{N\times 2-\left[\left(nIC+n\prime LS\right)\times 2\right]}{N\times 2}\times 100,$$

where *N* is the total number of the tested samples, *n*IC is the total number of samples that showed intermediate call (IC) and *n*ʹLS is the total number of samples with low signal.

(3) $$CADR\left(\%\right)=\frac{N\times 2-\left(nIC+n\prime LS\right)}{N\times 2}\times 100.$$

This formula was used when the warning message for a blood group was for only one allele and the $s^{nd}$ allele gave a correct result.

(4) $$CoR\left(\%\right)=\frac{N\times 2-\left[\left(nIC+n\prime LS\right)\times 2\right]-n\prime \prime DA}{N\times 2-\left[\left(nIC+n\prime LS\right)\times 2\right]}\times 100,$$

where *N*, *n*IC, *n*ʹLS, and *n*ʹʹDA show the total number of tested samples, the total number of samples with intermediate calls, the total number of samples with low signal, and the total number of alleles with discrepancy versus predicted results based on serologic results, respectively.

### 2.2 Serological test

All the blood samples collected for the BioArray study (BA study) including platelets and reference panels were phenotyped serologically for Fy^a^ and Fy^b^. The blood group phenotyping was performed based on the technical recommendations from suppliers for each monoclonal antibody/antiserum. All the samples with a discrepancy of serological results for Fy^b^ were re‐phenotyped using two different polyclonal anti‐Fy^b^ to check and confirm the donors' phenotype results.

### 2.3 In‐house PCR‐SSP

To produce large amounts of a specific DNA fragment of defined length and sequence from a small amount of a template, the PCR can be used as an in‐vitro method.^21^, ^22^ In this study, we used optimized and validated PCR‐SSP. *Fy* and *human growth hormone (HGH)* primers were synthesized by MWG at a scale of 0.01 µM. All the stock and working primers were made up at the concentration of 100 and 2 µM, respectively. Additionally, each PCR experiment included an internal positive control (HGH) and a negative DNA control known as No Template Control (NTC) which the DNA sample was replaced by $dH_{2}O$.^23^

### 2.4 Commercial PCR‐SSP kits

These kits are produced by different companies and are expected to perform more robustly than in‐house protocols since they usually pass different stages of the validation process and quality controls before being introduced to the market. In this study, the commercial PCR‐SSP kit (READY GENE KKD PCR‐SSP Kit) was used as an additional method for confirmation and verification of some achieved results.

### 2.5 Automated DNA sequencing

Sequencing of cloned/cleaned up DNA products for the BioArray study was performed by DNA Sequencing and Services (MRCPPU, College of Life Sciences, University of Dundee, Scotland) using Applied Biosystems Big‐Dye Ver 3.1 chemistry on an Applied Biosystems model 3730 automated capillary DNA sequencer. The sequencing results of the *FYX* allele‐positive donors were compared with the outcomes of *FYX*‐associated mutations in white American donors.^24^

## 3 RESULTS

The serological blood group phenotyping and the HEA *BeadChip™* genotyping results are as follows:

### 3.1 Haemagglutination blood group phenotyping results

The haemagglutination blood group phenotyping results are presented in Table 1.

**Table 1:** Summary of Duffy blood group phenotypes and actual genotype results from hemagglutination and microarray typing of 222 platelet and reference panel donors from Aberdeen Regional SNBTS.

Blood group	Duffy								
Associated SNPs tested by BioArray BeadChips	*FYA/B*			FY‐265			GATA		
Predicted genotypes based on serologic results	Fy^a^/Fy^a^	Fy^a^/Fy^b^	Fy^b^/Fy^b^	NA			NA		
Number phenotyped (Serology)	39	95	88	NA			NA		
BioArray genotypes	AA	AB	BB	AA	AB	BB	AA	AB	BB
Number genotyped (BioArray)	32	102	88	210	12	0	220	2	0
Number of samples with discrepancies	7/222			NA			NA		
Allele discrepancy rate versus serologic results	7/444			NA			NA		
CoR (%) between predicted genotypes from haemagglutination versus genotyping	98.42			NA			NA		

Abbreviations: NA, not applicable; SNP, single‐nucleotide polymorphism.

### 3.2 Microarray blood group genotyping results

The results of the blood group genotyping, the determined allele frequencies, overall ADR for the Duffy blood group as well as CoR between predicted genotypes from haemagglutination and genotyping results of microarray assay are shown in Table 1.

In the present study, 0 of 222 samples showed IC or LS. ADR and CADR were 100%.

As it is shown in Table 1, genotype discrepancies versus serological phenotyping outcomes occurred in seven donors possessing Fy^b^ (*n* = 7; BA048, BA078, BA115, BA141, BA155, BA162, and RP35, which are based on the sample ID). As mentioned before, the discrepant samples were re‐phenotyped and re‐genotyped and this will be discussed in the following section.

### 3.3 Re‐phenotyping of discrepant samples

The re‐phenotype results with different antisera were in good agreement with the original serological results that were obtained using antisera from Alba Bioscience with slight differences in their grades of hemagglutination (±1 grade variation of reaction at the most).

### 3.4 Re‐genotyping of discrepant samples

To validate the results obtained by HEA BeadChip assays for the *FYB* discrepant samples, the Inno‐TRAIN KKD PCR‐SSP kit was used as one of the confirmatory tests. A summary of the results is presented in Table 2.

**Table 2:** Summary of the results from KKD PCR‐SSP typing for seven discrepant and a control patient sample with SCD (MA).

Block specificity	Duffy			
Reaction No.	5	6	7	8
Glycoprotein	Fy^a^	Fy^b^ Fy^b^ weak	Fy null	Fyb weak
Specificity of the products	*FYA*	*FYB*	*FY* * *Null01*	*FYX*
PCR‐SSP Product size (bp)	720	720	720	187
Presence of 434 bp Internal control band**	+	+	+	+
BA 048	+	+	−	+
BA 087	+	+	−	+
BA 115	+	+	−	+
BA 141	+	+	−	+
BA 155	+	+	−	+
BA 162	+	+	−	+
RP 35	+	−	+	−
MA	−	−	+	−

*Note*: The positive (+) and negative (−) signs indicate the appearance and absence of a PCR product band.

* This sometimes tends to show very weak bands, however, the genuine reactions are clearly distinguished by the strength and intensity of the bands.

** This indicates that for all the samples this internal control band appeared, indicating the PCR protocol had not failed and there was enough gDNA for amplification.

Moreover, the in‐house PCR‐SSP has been also used as a confirmatory test and its results are in good agreement with the results of HEA BeadChip assays and commercial conventional PCR‐SSP kits.

### 3.5 DNA sequencing of discordant samples

The results of DNA sequencing for discordant donor samples (BA048, BA087, BA115, BA141, BA155, BA162, RP35) and a control patient sample with Sickle Cell Disease (SCD) (MA), observed mutations, and also the amino acid substitutions have been presented as first 8 samples in Table 3.

**Table 3:** Summary of final results for all Duffy DNA sequencing assays.

Region specificity		GATA	Fy^a^/Fy^b^		Fy^x^				
Number (Sample ID)	Allele	Nt −67	Nt 125	Aa 42	Nt 265	Aa 89	Nt 298	Aa 100	Confirmed Genotype
6 (BA048, BA087, BA115, BA141, BA155, BA162)	1st	T	G	Glycine	C	Arginine	G	Alanine	*FYA*
	2nd	T	A	Aspartic acid	T	Cysteine	A	Threonine	*FYX*
1 (RP35)	1st	T	G	Glycine	C	Arginine	G	Alanine	*FYA*
	2nd	C	A	Aspartic acid	C	Arginine	G	Alanine	*FY*
1 (MA)	1st	C	A	Aspartic acid	C	Arginine	G	Alanine	*FY*
	2nd	C	A	Aspartic acid	C	Arginine	G	Alanine	*FY*
4 (BA017, BA083, BA128, BA154)	1st	NT	A	Aspartic acid	C	Arginine	G	Alanine	*FYB*
	2nd	NT	A	Aspartic acid	T	Cysteine	A	Threonine	*FYX*
2 (BA042, BA055)	1st	NT	A	Aspartic acid	C	Arginine	A	Threonine	*FYB*
	2nd	NT	A	Aspartic acid	T	Cysteine	A	Threonine	*FYX*
Denotes missense mutations			Denotes an SNP		Denotes missense mutations without a significant phenotypic change				

*Note*: To avoid confusion, standard three‐letter codes have been used for all correspondence Amino acids in this table.

Abbreviations: A, Alanine; Aa, Amino acid; C, Cysteine; D, Aspartic acid; G, Glycine; MA, a homozygous *GATA* positive control sample; NT, not tested; Nt, nucleotide; R, Arginine; T, Threonine.

To investigate the GATA mutation at the promoter region, the positions of nucleotide 125 G > A (Nt 797), which encoded the *FYA/FYB* alleles, and two nucleotide substitutions at 265 C > T and 298 G > A (Nt 937 and Nt 970, respectively) that are known as mutations related to *FYB/FYX*, the entire amplified *FY* gene for all eight samples has been sequenced with different forward and reverse primers. As shown in Table 3, the sequence analysis of exon 2 in all of these samples shows the existence of a'Gʹ nucleotide in one of the alleles at position 125. The sequencing results for the rest of the coding region for both exons (exon 1 and exon 2) and the promoter regions were similar to the previously reported *FY* gene sequences deposited in GenBank from normal Duffy individuals. Furthermore, for six of these eight samples, the sequence analysis of exon 2 in the 2^nd^ alleles indicated an'Aʹ nucleotide at position 125 with two extra nucleotide substitutions, T for C at position 265, and G for A at position 298 that these mutations are the specification of the *FYX* allele. Moreover, sample RP35 was identified as heterozygous for the GATA mutation (see Table 3).

Of 12 donors with AB genotype FY‐265 by microarray (see Table 1), the six samples with Fy(a^−^ b^+^) phenotypes with no discrepancy between the serological and microarray genotyping results, were examined by direct DNA sequencing as a complementary investigation. The summary of final direct DNA sequencing results for these samples is listed in Table 3 (the final six samples). All of these six samples are homozygous *FYB*, due to the presence of the polymorphic site (125 G > A) which is the characteristic of the Duffy phenotype. Moreover, the molecular presence of the polymorphic sites 265 C > T and 298 G > A that are indicative of the *FYB/FYX* indicates that samples BA128, BA083, BA154, and BA017 are heterozygous *FYB/FYX* genotypes for both polymorphic markers due to the presence of two simultaneous peaks at both positions. However, the BA042 and BA055 samples are heterozygous for *FYB/FYX* at position 265 but homozygous mutant for nucleotide 298. Consequently, in the present study, the existence of 265 C > T mutation reduced antigen expression on the surface of cells leading to negative serological results. It has to be noted that the 265 C > T missense mutation always occurs with 298 G > A mutation while the 298 G > A missense mutation can be found alone. However, the 298 G > A mutation without 265 C > T mutation can not decrease the antigen expression and lead to positive serological results. 50% of 12 *FYX* allele‐positive donors were phenotyped as Fy (a^+^ b^−^) and the remaining as Fy (a^−^ b^+^) by the serological test. Whereas, by the HEA BeadChip method through recognition of mutation at nucleotide 265, heterozygous *FYA/FYB* and homozygous *FYB/FYB* were genotyped, respectively. The sequencing results of the 12 samples were compared with the outcomes of *FYX*‐associated mutations in 47 white American donors (Table 4).^24^

**Table 4:** *FYX*‐associated polymorphisms in the Scottish and White American.

Genotype	*FYX*‐associated mutations		Number of donors^a^	
*FY*	265 C > T	298 G > A	Scottish	American
*FYA/FYB*	wt/wt	wt/m	0	4
	wt/m	wt/m	6	2
	wt/m	m/m	0	0
*FYB/FYB*	wt/wt	wt/m	0	9
	wt/m	wt/m	4	0
	wt/m	m/m	2	0

Abbreviations: m, mutated allele; wt, wild type allele.

^a^ The number of donors found with the *FYX*‐associated mutations within 222 genotyped Scottish donors' samples in this study and between 47 investigated White American donors.

## 4 DISCUSSION

In the present study, in 12 of 222 Scottish donors (5.41%), one *FYX* allele was discovered (see Table 3). The frequency of the *FYX* allele was estimated to be 0.0270%. This is in agreement with a study conducted by Murphy et al.,^14^ where they examined 109 serologically Fy (a^+^ b^−^) typed samples of Scottish donors using the PCR‐RFLP method. Due to the high incidence of the discrepancy between *Fy* ^*b*^ phenotyping and genotyping results, they concluded that the frequency of *Fy* ^*x*^ was more than previously reported 0.015%.^13^ Thirteen of 109 samples (11.92%) investigated by Murphy et al. were genotyped as *FYA/FYB*, while in the present study, 6 of 39 (15.38%) serologically typed Fy(a^+^b^−^) samples were genotyped as *FYA/FYB* with two *FYX*‐associated mutations. The donors who participated in the present study, their parents, and their grandparents were Caucasians. Hence, this is the highest prevalence of *FYX* that has been reported in the Scottish population.

Several blood groups exist on red cell membrane which are grouped into different systems.^25^ After the ABO system, Duffy is the second most significant blood grouping system.^26^ Fy^a^ and Fy^b^ are the two major antigens of the Duffy blood group system.^27^ The substitution c.265 C > T attenuates Fy^b^ antigen leads to the Fy^x^ phenotype.^28^ The frequency of the *FYX* allele varies greatly in different populations. Hongfongfa et al.^29^ in a study on malaria patients in Thailand, did not find the mutation of the *FYX* at nucleotide position 265 in 167 *FYB* negative samples. Van Alsten et al.^30^ reported one patient with Fy^x^ in 3537 non‐Hispanic white participants. Sheppard and colleagues^31^ in a study on a Hispanic population, reported a frequency of 1.2% for the Fy^x^ phenotype. Chown et al. estimated a frequency of 2% for the *FYX* allele in a Caucasian population.^8^ Frequencies of 0.025 in Swedish blood donors and 0.013 in Australian donors have also been estimated for the *FYX* allele.^19^, ^32^, ^33^

As mentioned earlier, the molecular basis of the *FYX* allele is due to the existence of two mutations, including the substitution of C to T at nucleotide 265 and G to A at nucleotide 298.^9^, ^34^ These mutations lead to the substitution of Arginine to Cysteine at position 89 and Alanine to Threonine at position 100, respectively. Instability and reduction of Fy^b^ protein in the cell surface result from the mentioned amino acid changes.^35^, ^36^ According to Table 3, in two donors (BA042 and BA055) the missense mutation at position 298 G > A was found in just one allele, however, both *FYX* ‐associated mutations were in the other allele. Since these two samples were Fy^b+ve^ by the usual serologic typing, missense mutation at 298 position alone does not seem adequate to change the Fy^b^ expression at the significant level and prevent the other *FYB* allele from being read. The existence of this mutation has been reported in 33% of Caucasians.^32^ In the present study, in contrast to the 265 C > T mutation, the 298 G > A polymorphism did not lead to instability of Fy^b^ protein antigen and reduction of antigen expression in erythrocytes.

## 5 CONCLUSION

This study aimed to investigate the *FYX* allele frequency in the Scottish population. The frequency of the *FYX* allele was estimated to be 0.0270% which is, to the best of our knowledge, the highest frequency reported so far in the Scottish population. However, further studies with larger sample sizes are recommended to evaluate the *FYX* allele frequency more precisely.

## AUTHOR CONTRIBUTIONS

**Sylvia Armstrong‐Fisher**: Conceptualization. **Stan Urbaniak**: Conceptualization. **Arian Karimi Rouzbahani**: Writing—original draft. **Mona Hemmati Chegeni**: Formal analysis. **Golnaz Mahmoudvand**: Writing—original draft. **Ali Mohammad Varzi**: Conceptualization; supervision; formal analysis; writing—review and editing.

## CONFLICT OF INTEREST STATEMENT

The authors declare no conflict of interest.

## TRANSPARENCY STATEMENT

The lead author Ali Mohammad Varzi affirms that this manuscript is an honest, accurate, and transparent account of the study being reported; that no important aspects of the study have been omitted; and that any discrepancies from the study as planned (and, if relevant, registered) have been explained.

## Data Availability

The data that support the findings of this study are available from the corresponding author upon reasonable request.
